# Optimising natural rubber recycling capability using combinations of copper(ii) ion and phosphine inhibitors

**DOI:** 10.1039/d6su00080k

**Published:** 2026-08-18

**Authors:** Thomas Griggs, Anureet Kaur, Keizo Akutagawa, Biqiong Chen, James J. C. Busfield

**Affiliations:** a School of Engineering and Materials Science, Queen Mary University of London Mile End Road London E1 4NS UK j.busfield@qmul.ac.uk; b Department of Chemistry, University of Liverpool Crown Street Liverpool L69 7ZD UK

## Abstract

Previous work demonstrated that controlled inhibition of disulphide metathesis using copper(ii) methacrylate (CuMA) can enable high recycling efficiency in conventional (CV) and semi-efficient (SEV) cure systems. However, CuMA's high cost and limited commercial availability restrict its industrial application. This study investigates triphenylphosphine (TPP) as a more accessible inhibitor, used alone or in combination with CuMA, to achieve comparable recycling performance at reduced cost. Cure characteristics were evaluated using Moving Die Rheometer analysis, and mechanical properties were assessed through tensile testing to quantify recycling efficiency. Results show that TPP alone provides moderate recyclability, while TPP–CuMA blends deliver efficiencies similar to pure CuMA in CV systems and superior performance in SEV systems. These findings highlight a commercially viable approach to recycling sulphur-cured NR compounds, reducing reliance on virgin rubber and supporting sustainable manufacturing.

## Abstract 2

A combination of copper(ii) ion and phosphine allows for highly recyclable crosslinked natural rubber systems.

## Graphical abstract

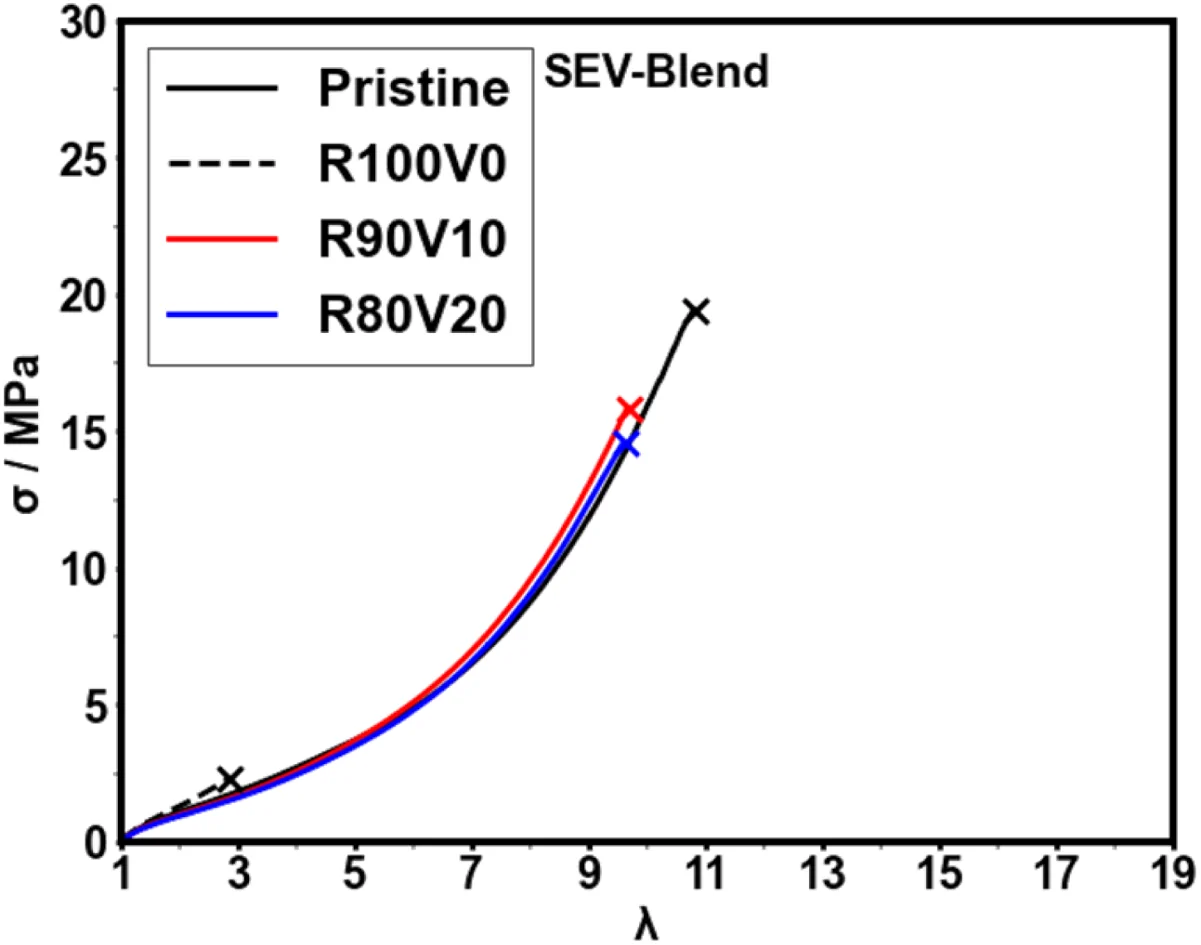

**Sustainability spotlight**

The work within this paper seeks to progress previous work on recyclable crosslinked rubber materials; it seeks to target multiple UN sustainability goals, including Goal 9, Industry, innovation and infrastructure, through allowing for sustainable industrialisation, particularly through allowing for circular economy natural rubber products. Goal 12, Responsible consumption and production, is also targeted through increasing recycling of rubber waste, and to minimise the need for new rubber to be harvested. Finally, goal 13, Climate action, is targeted through reducing the carbon footprint of natural rubber products, through reutilisation.

## Introduction

Tyres are generally produced using a range of different elastomer compounds depending on their location in the tyre and their function. Natural rubber (NR) and styrene butadiene rubber (SBR) are the most widely adopted polymers incorporated into these compounds. Other materials such as fillers or metal reinforcements are also added to reinforce the rubber and to maintain structural integrity of the tyre. The wide range of different applications for tyres also results in large differences in the composition of elastomer compounds, with NR typically being more prevalent in truck and aircraft tyres, whereas passenger car tyres have a higher proportion of SBR in their composition.^1^

While several products utilise NR, tyres are the most common application using about 70% of the total global production of roughly 15 million tonnes in 2024.^2^ To meet the highly demanding specifications of tyre applications, both NR and SBR are crosslinked (or cured) during a moulding phase.

This is typically done at elevated pressures and temperature in the presence of elemental sulphur and other crosslinking ingredients mixed into the rubber. For certain high temperature applications more thermally stable crosslinks such as carbon linkages formed using a peroxide cure system are adopted.

A clear downside to the curing process is the formation of chemically permanent crosslinks between the polymer chains, which severely limits the recycling of the elastomer compounds. This often results in the end-of-life recovery being restricted to energy recovery or pyrolysis,^3,4^ rather than through reutilisation of the higher value polymer/filler materials directly. These recycling routes not only release significant amounts of harmful chemicals or gases they also only recover about 25% the input energy.^5^ Attempts have been made to recycle the polymer directly, however this is often done through downcycling ground up tyres into applications such as playground floors or sports fields, or additionally as a filler incorporated into construction materials.^6,7^ Specialised milling processing, chemical, ultrasonic or microwave breakdown methods have demonstrated promise in producing reprocessable cured materials.^8–11^ The drive to increase the amount of elastomer compounds that are recycled back into high value products such as tyres is now inescapable.

The only alternative to meet the growing demand for NR would be to increase the amount of rubber plantations rather than increasing the recycled content on the market. However, this would have a negative environmental impact through increased deforestation, producing a further reduction in biodiversity.^12^ There are also issues about security of supply if any of the NR producing nations were to be hit for example by the spread of a fungal infection.^12^

Depending on the intended usage of the rubber product, different sulphur vulcanisation systems are developed during the curing process. The rank or length of the sulphur crosslink depends upon the ratio of accelerators to sulphur. Conventional vulcanisation (CV) compounds have a low ratio of accelerator to a high sulphur content. This produces predominantly poly- or disulphide crosslinks.

This results in materials with high toughness, but poor thermochemical stability and therefore poor ageing properties. To increase the polymer stability for higher temperature application, an efficient vulcanisation system (EV) is used.

This requires the opposite ratio of high accelerator together with a low sulphur ratio. In an EV system, the predominant crosslinks are monosulphides, which are more thermally stable, however a reduction in resulting compound strength is seen. The semi-efficient vulcanisation system (SEV), formed using a more balanced ratio of sulphur to accelerator, is widely adopted in practice, with more abundant disulphide crosslinks present. SEV provides a balance of the high toughness from CV and the thermal stability and ageing resistance of the EV.

Previous studies on both unfilled CV system and unfilled SEV system have demonstrated that using copper(ii) methacrylate (CuMA) as an inhibitor can facilitate the recycling of these compounds under specific conditions.^13,14^ This research suggests that various crosslinking systems can be employed alongside chemical inhibition of disulfide metathesis. However, CuMA is not commercially available and remains costly, which limits its practical application. The work reported in this paper investigates a different more commercially viable chemical inhibitor system containing triphenylphosphine (TPP) with and without CuMA, to evaluate a lower cost alternative inhibitor system to support potential elastomer compound recycling. In the current work, rheology was carried out to understand the curing characteristics, quasi-static tensile testing was done to evaluate maximum strain and strength and to provide a comparison between pristine and recycled strain and strength values for both the CV and SEV curing system.

Previous investigations indicated that a molar ratio of 1 : 4 between CuMA and the total number of disulphide bonds provides a good recycling efficiency in the CV system.^13^ A 1 : 4 ratio was found not to be optimal for the SEV system, testing lower ratios showed that a ratio of 1 : 16 was the most effective.^14^

## Results

To understand the curing characteristics of the CV systems and to see how they compared to the previously reported CV CuMA 1/4 system,^13^ Moving Die Rheometer (MDR) analysis was carried out at 150 °C for 30 min (Fig. 1). Testing showed the cure characteristics were dependent upon which inhibitor system was utilised.

**Fig. 1:**
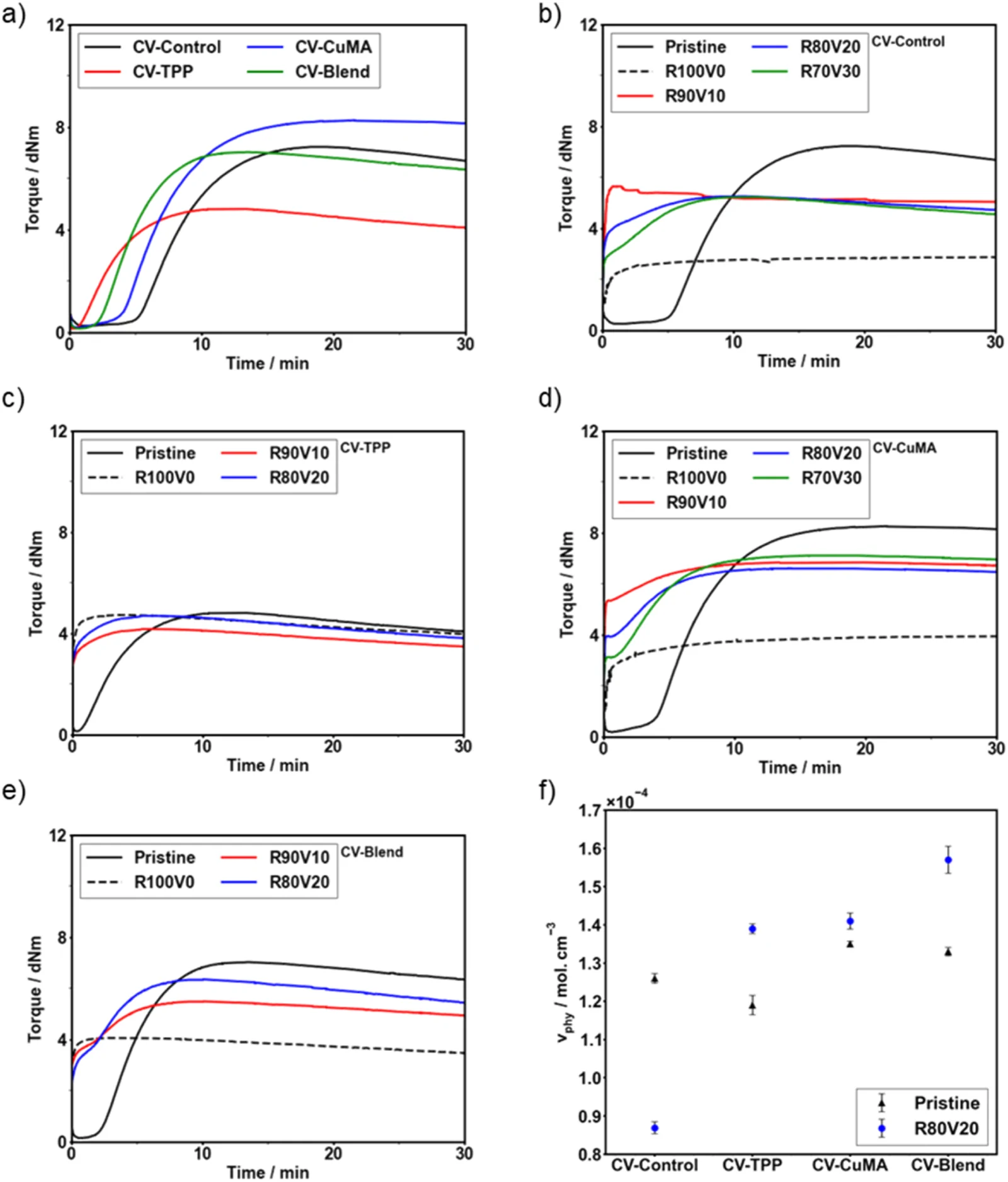
MDR analysis at 150 °C for 30 min. (a) comparison between CV-control (solid black line), CV-CuMA (solid blue line), CV-TPP (solid red line) and CV-blend (solid green line) curing characterisation of recycled compounds at 150 °C for 30 min. MDR analysis comparison between pristine (solid black line) and recycled compounds R90V10 (solid red line), R80V20 (solid blue line) and R70V30 (solid green line) for (b) CV-control, (c) CV-TPP, (d) CV-CuMA, (e) CV-blend, (f) crosslink density for pristine and R80V20 for CV formulations.

Firstly, the addition of any inhibitor reduced the scorch time of the system (SI Table 1), with CV-TPP showing the shortest time to cure, followed by CV-Blend, then CV-CuMA with CV-Control having the longest processing window. Following the same hierarchy as scorch time, the maximum torque values seen for the systems are changed significantly by the presence of the different inhibitors. CV-TPP again showing the lowest torque values indicting a suppression of the total number of crosslinks, and CV-CuMA showing the highest maximum torque. An additional observation lies in the more stable plateau in the cure characteristics of CV-CuMA, with both the control and the other two inhibitor systems showing reversion, while pure CuMA remains stable up to 30 min.

Evaluation of the recycled compounds for CV-Control, CV-TPP, CV-CuMA, and CV-Blend was also undertaken using the MDR analysis at 150 °C for 30 min, to examine scorch time, torque recovery, and reversion characteristics (Fig. 1b and e). Each of the compounds showed a much reduced or non-existent scorch time in any of the recycled materials, attributed to a partially crosslinked structure remaining in the recycled content. Each compound showed a small increase in torque during the first few minutes of curing, attributed to the virgin materials or inhibited disulphide radicals creating additional, or reforming, crosslinks in the material. Despite this, in each case, the maximum torque achieved by the pristine material was not met by the recycled materials. As with the pristine materials, CV-CuMA showed good reversion control, even in the recycled materials, whereas CV-TPP and CV-Blend all showed some minor reversion. CV-Blend shows an interesting initial cure, with a small increase, followed by a small inflexion before then increasing again up to the maximum achieved torque value, this is assumed to be a result of the two different inhibitors having slightly different activation energies, resulting in two different but overlapping cure rate reactions.

Similar crosslink densities (*ν*_phy_) can be seen for each of the pristine formulations (Fig. 1f), however CV-TPP shows a minor reduction in the crosslink density. A larger difference is seen in the recycled compounds, where CV-control shows a significant reduction in the crosslink density, suggesting that with no inhibitor there is an uncontrolled breakdown of the system, resulting in poor properties. CV-CuMA on the other hand shows very similar crosslink densities between pristine and the recycled compound, suggesting it is efficiently maintaining the crosslink structure during the breakdown and reformation steps in the recycling. Both CV-TPP and CV-blend show a slight increase in the crosslink density, which could be a result of the majority of disulphide crosslinks being maintained by the inhibitor, with potential new ones forming through reduction of polysulphides to a lower ranking.

Uniaxial tensile tests were carried out to measure the mechanical behaviour of each compound. Fig. 2 shows the representative curve for each sample. The addition of CuMA causes an upturn in the modulus at larger strains, whereas the addition of TPP slightly reduces the modulus, while significantly increasing the strain ratio. Reflecting the rankings of the maximum torque values seen in the MDR curves, this would suggest a lower crosslinking density is achieved within the TPP compounds. CV-control showed the lowest overall properties, followed by CV-CuMA, then CV-TPP and finally CV-blend (Table 1). Another parameter that can be considered is the strain energy density at break for the compounds, where a slightly different ranking can be seen. CV-TPP shows the highest energy to break at 70.7 MJ m^−3^, followed by CV-blend, then CV-CuMA and finally CV-control with values of 66.7, 63.8 and 44.4 MJ m^−3^ respectively. As seen in previous papers compounds containing CuMA show a significant improvement in their recyclability,^13,14^ especially when examining the *σ*_max_ of the materials. CV-control and CV-TPP showed slightly lower recycling efficiencies as well as greater deviation from the pristine curve at the higher strains. However, the blend of TPP with CuMA gives enhanced performance of the recycled compounds, comparable to CV-CuMA, and in both cases, the recycled stress–stretch ratio curves follow the pristine curve very closely. R80V20 showed good recovery of properties, with the strain ratio showing higher efficiencies than the stress at break. To better visualise the effectiveness of the inhibitor systems, the recycling efficiencies were also calculated (Fig. 3). CV-TPP showed worse performance with a recycling efficiency for stress (*η*_*σ*_) of 75% and a recycling efficiency of strain ratio (*η*_*ε*_) of 36%, very similar to the values seen for CV-Control (Fig. 3a and b) with a *η*_*ε*_ of 73% and a *η*_*σ*_ of 34% for R80V20. This suggests that TPP on its own cannot produce recyclable materials effectively, however, as noted from the tensile data, the addition of a very small addition of CuMA significantly enhances its performance as an inhibitor. R80V20 for CV-Blend showed a *η*_*ε*_ of 96% and a *η*_*σ*_ of 84%, similar to CV-CuMA, with values of 94% and 89% for *η*_*ε*_ and *η*_*σ*_ respectively. For both CV-Blend and CV-TPP, a recyclate ratio of 50% was used as this could be considered more industrially viable, as well providing insight on achievable efficiencies. CV-TPP showed lower properties, with a *η*_*σ*_ of 65% and a *η*_*ε*_ of 81% for R50V50. Conversely, CV-Blend, within experimental error, completely recovered its properties with a *η*_*σ*_ of 95% and a *η*_*ε*_ of 97%.

**Fig. 2:**
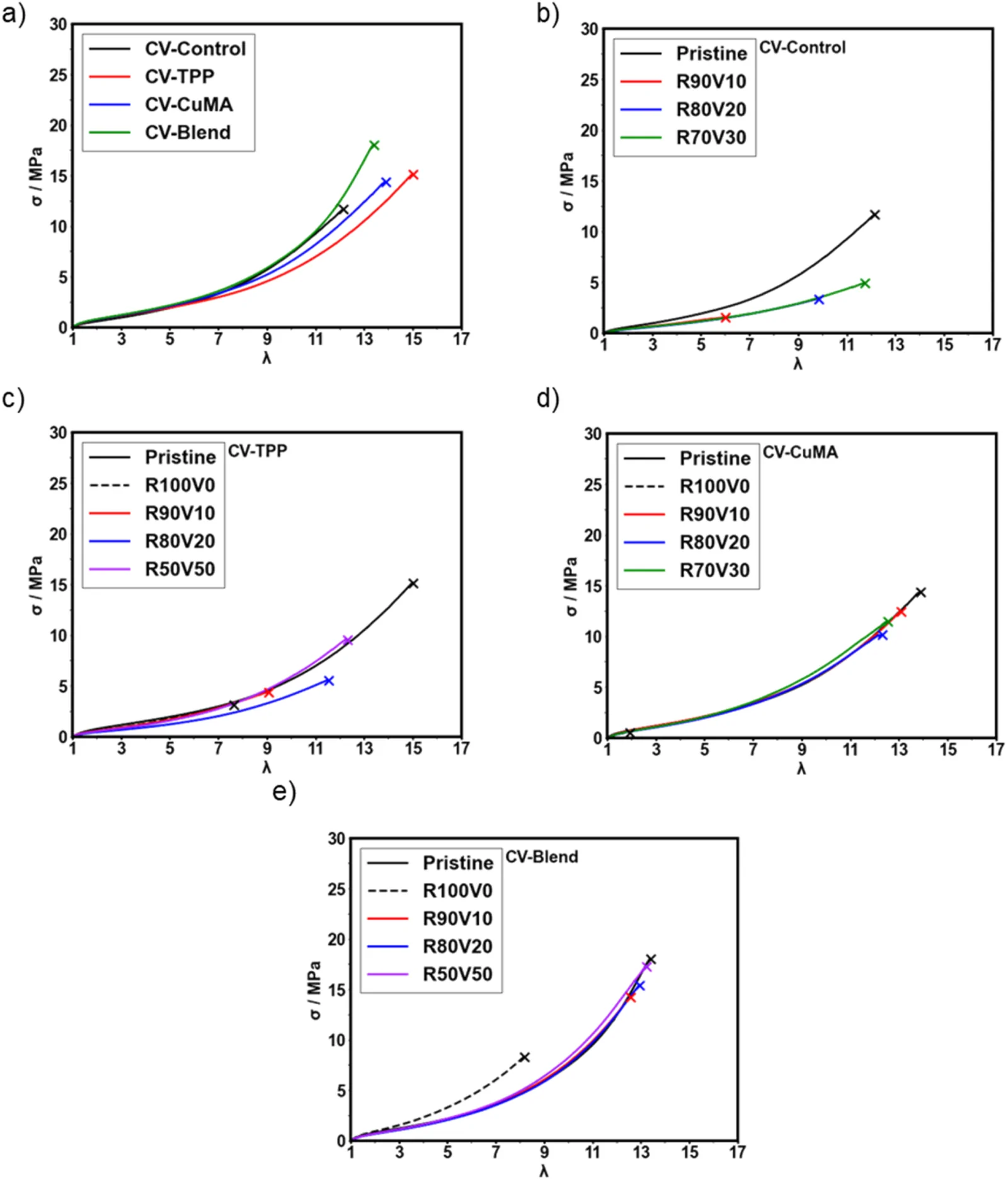
Stress–strain curves from tensile test. (a) Comparison between CV-control (solid black line), CV-CuMA (solid blue line), CV-TPP (solid red line) and CV-Blend (solid green line) – comparison between typical tensile curves of pristine (black line), R100V0 (dashed black line), R90V10 (solid blue line), R80V20 (solid red line), R70V30 (solid green line) and R50V50 (solid purple line) for (b) CV-control, (c) CV-TPP, (d) CV-CuMA, and (e) CV-blend.

**Table 1:** Maximum *λ* and *σ* values for CV pristine compounds (left) and R80V20 (right)

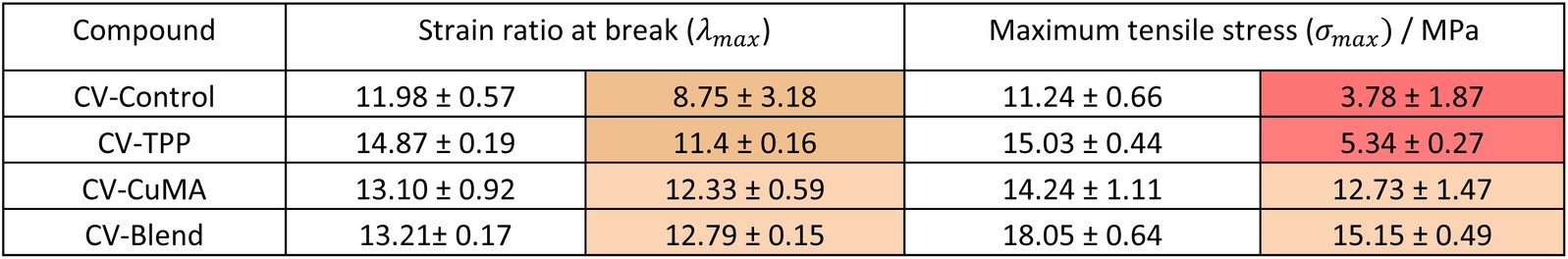

**Fig. 3:**
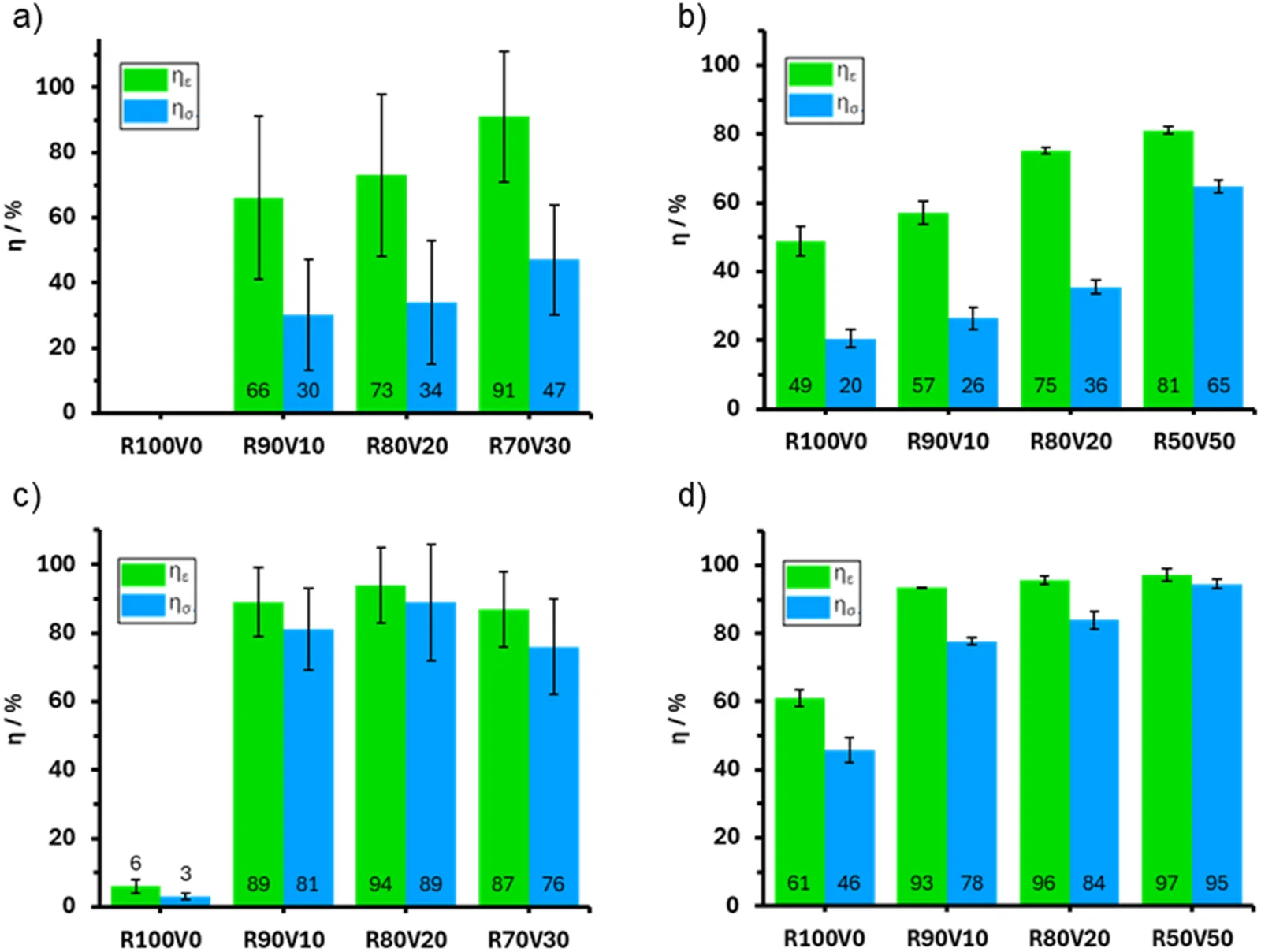
Recycling efficiency in terms of stress and strain of recycled compounds. Results for: (a) CV-control, (b) CV-TPP, (c) CV-CuMA, and (d) CV-blend. Green bars for *η*_*σ*_, blue bars for *η*_*ε*_, standard deviation in solid black line.

For CV-CuMA and CV-blend, the slight reduction in properties despite following the pristine curve closely could be a result of an increase in intrinsic flaw size through the incorporation of recycled rubber, resulting in lower overall strength.^15^ CV-blend showed quite notable recovery of properties even at a 100% recycled content, suggesting significant potential for fully recycled products with lower strength requirements.

The curing characteristics of the SEV compounds were measured using MDR analysis (Fig. 4). As with the CV system, the addition of inhibitor systems reduces the scorch time of the compounds. The SEV ranking follow the same hierarchy as the CV ranking. After SEV-Control, SEV-CuMA shows the next longest scorch time, followed by SEV-blend and the shortest is for the SEV-TPP. For each SEV system, there is very little difference between the maximum torque values, at around 9 dN m, achieved for each of the materials with and without inhibitors, suggesting there is a smaller variation in the fully cured cross link densities within the SEV systems than was the case for the CV systems. When compared to the CV formulations, there is a general increase in crosslink density (Fig. 4f), which can be expected as the sulphur ranking shifts from polysulphide to more di- and mono-sulphides. In this case, the addition of the inhibitor produces a marked decrease in the crosslink density, with SEV-TPP showing the lowest pristine crosslinking density. SEV-control shows an increase in crosslink density after the recycling event, as compared to the dramatic reduction seen in CV-control. In the case of both SEV-TPP, SEV-CuMA and SEV-blend a small increase in the crosslink density is also seen, which could be attributed to the reduction of remaining polysulphide bonds into disulphide bonds (Fig. 5).

**Fig. 4:**
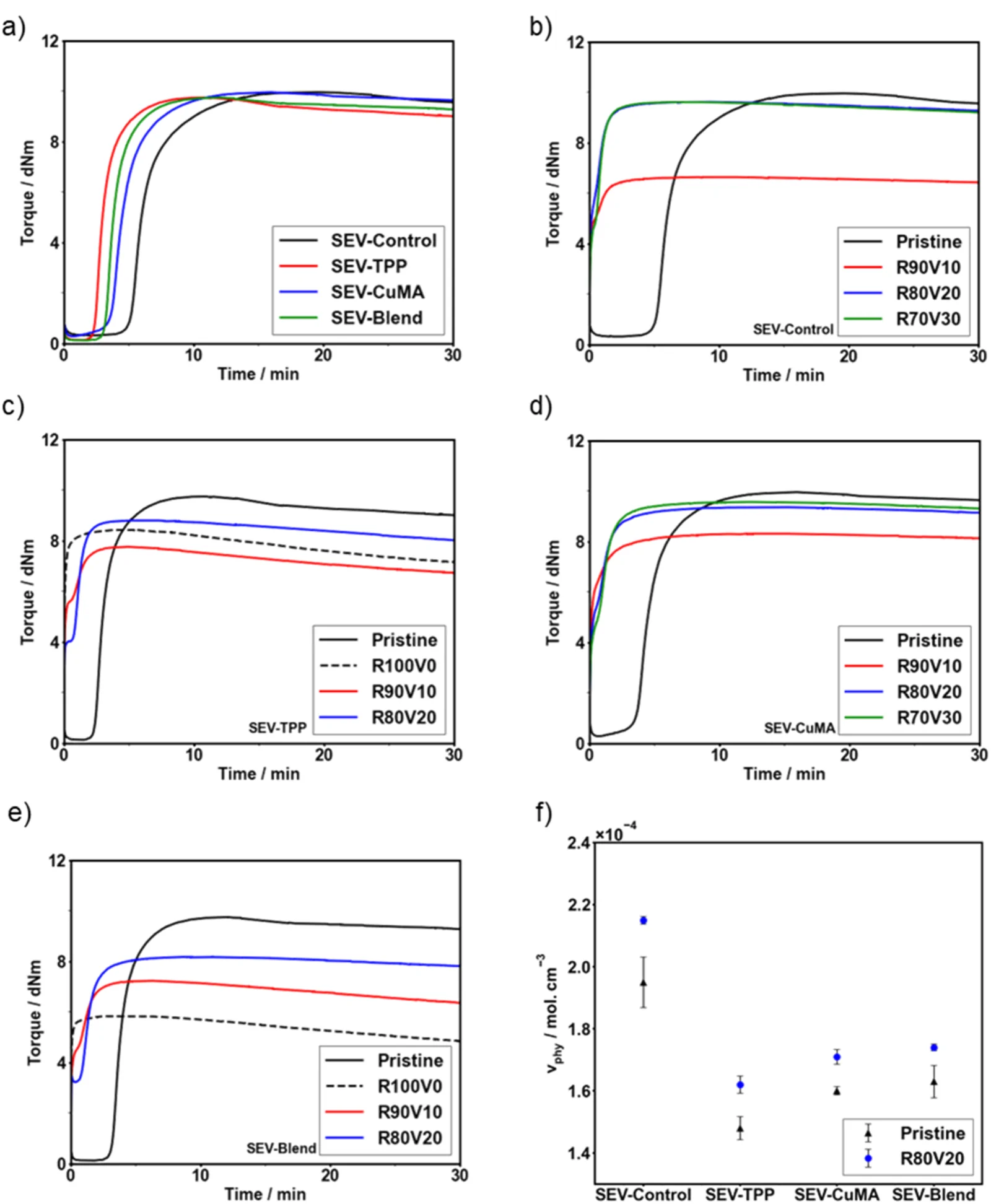
MDR analysis at 150 °C for 30 min. (a) Comparison between SEV-control (solid black line), SEV-CuMA (solid blue line), SEV-TPP (solid red line) and SEV-blend (solid green line) curing characterisation of recycled compounds at 150 °C for 30 min. MDR analysis comparison between pristine (solid black line) and recycled compounds R90V10 (solid red line), R80V20 (solid blue line) and R70V30 (solid green line) for (b) SEV-control, (c) SEV-TPP, (d) SEV-CuMA, (e) SEV-Blend, (f) crosslink density of pristine and R80V0 for SEV formulations.

**Fig. 5:**
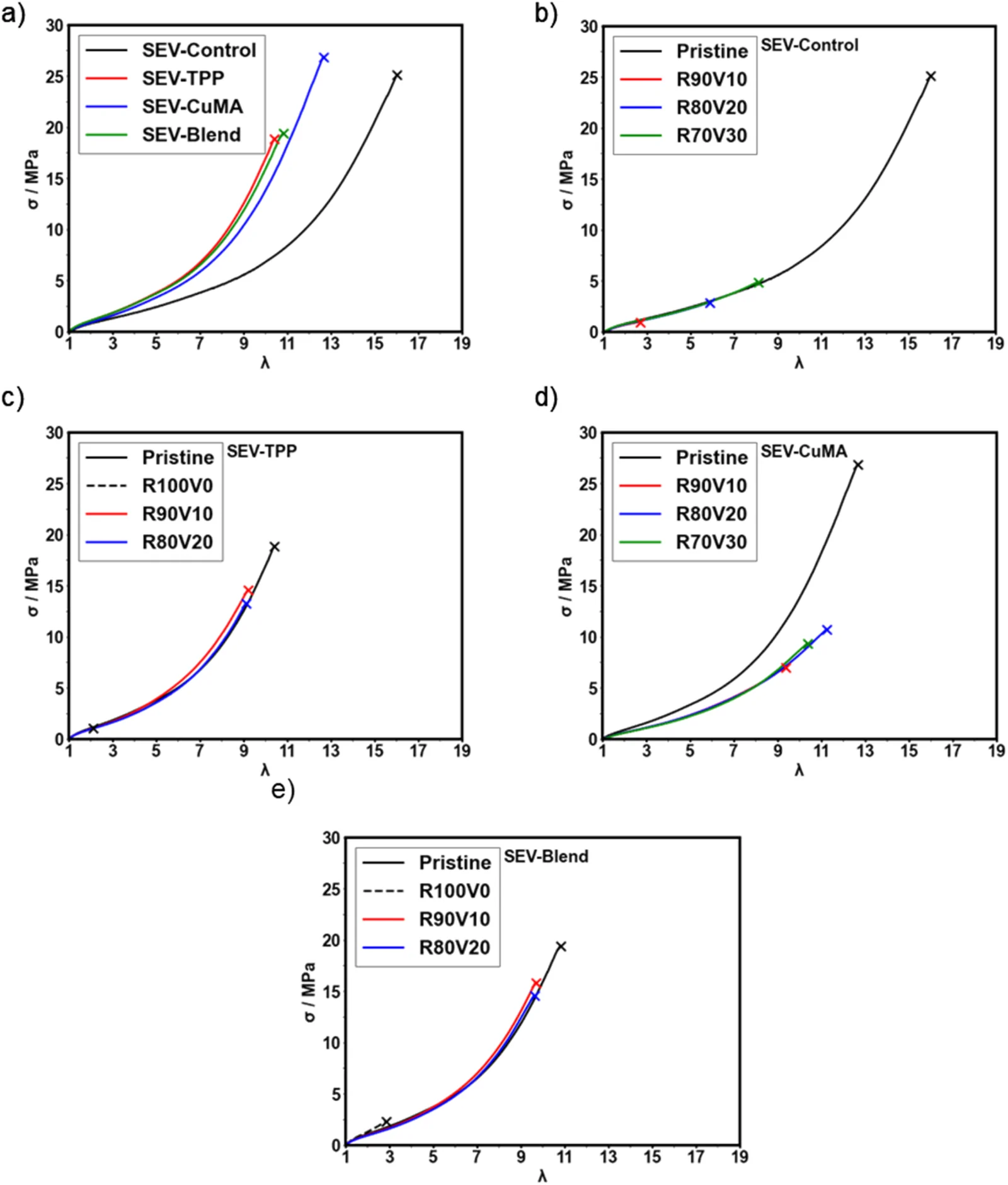
Stress–strain curves from tensile test. (a) comparison between SEV-control (solid black line), SEV-CuMA (solid blue line), SEV-TPP (solid red line) and SEV-blend (solid green line) – comparison between typical tensile curves of pristine (black line), R100V0 (dashed black line), R90V10 (solid blue line), R80V20 (solid red line) and R70V30 (solid green line) for (b) SEV-control, (c) SEV-TPP, (d) SEV-CuMA, and (e) SEV-blend.

Like the CV system (Fig. 2), the addition of CuMA increases the modulus of the materials, however, in this case, the addition of TPP shows a slight decrease in the strength of the material (Table 2). As with the CV systems, the energy to break for each compound can be calculated, both SEV-TPP and SEV-Blend show similar values to the CV compounds with values of 58 and 63 MJ m^−3^ respectively. SEV-Control and SEV-CuMA however show much higher energy to break with values of 111 and 96 MJ m^−3^.

**Table 2:** Maximum *λ* and *σ* values for SEV pristine compounds (left) and R80V20 (right)

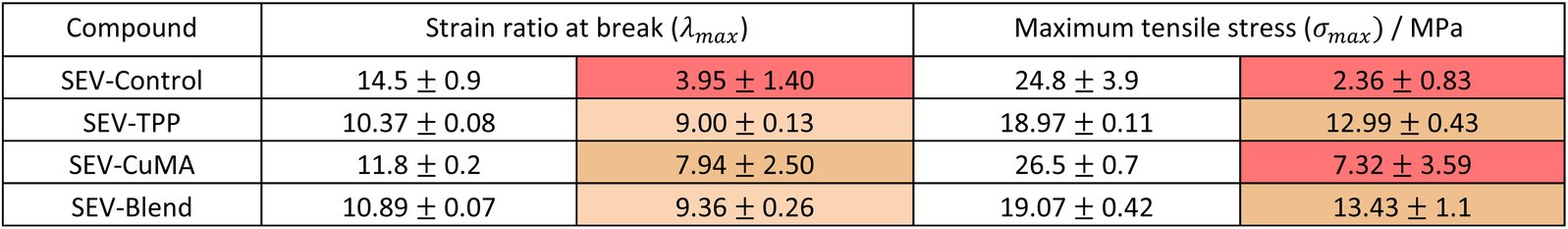

Once again, recycling efficiencies were calculated (Fig. 6). Unlike the CV system, there is very little notable recovery of properties for R100V0. In the CV system there is a potential for recycling to introduce new cross links through the breakdown of higher order sulphur crosslinks into new lower order crosslinks such as disulphides in CV. The less labile crosslinks in the SEV cure system are less able to undergo this type of transition. This shifting of the crosslink ranks may also provide insight into the reduction in efficiencies seen within the control sample, where a *η*_*σ*_ of 9.5% and a *η*_*ε*_ of 27.1% was seen, in comparison to 34% and 73%, respectively in CV-control. Additionally, SEV-TPP shows significant improvement to recycling efficiencies compared to CV-TPP, showing very similar results to SEV-Blend. Values for *η*_*σ*_ of 68% and 70%, and values of *η*_*ε*_ of 87% and 86% were seen in R80V20 for SEV-TPP and SEV-Blend respectively, with SEV-CuMA showing a lower efficiency with a *η*_*σ*_ of 21.7% and a *η*_*ε*_ of 60.1%. These values are an improvement to literature where Sombatsompop *et al.* achieved 50% recovery at 40% recyclate loading and Seghar *et al.* achieved 87% recovery at 20% recyclate loading.^16,17^ This all suggests that the combination of TPP and CuMA may enhance the inhibition of the disulphide bonds better than CuMA alone within a SEV system, where there are fewer bonds present when compared to a CV system.

**Fig. 6:**
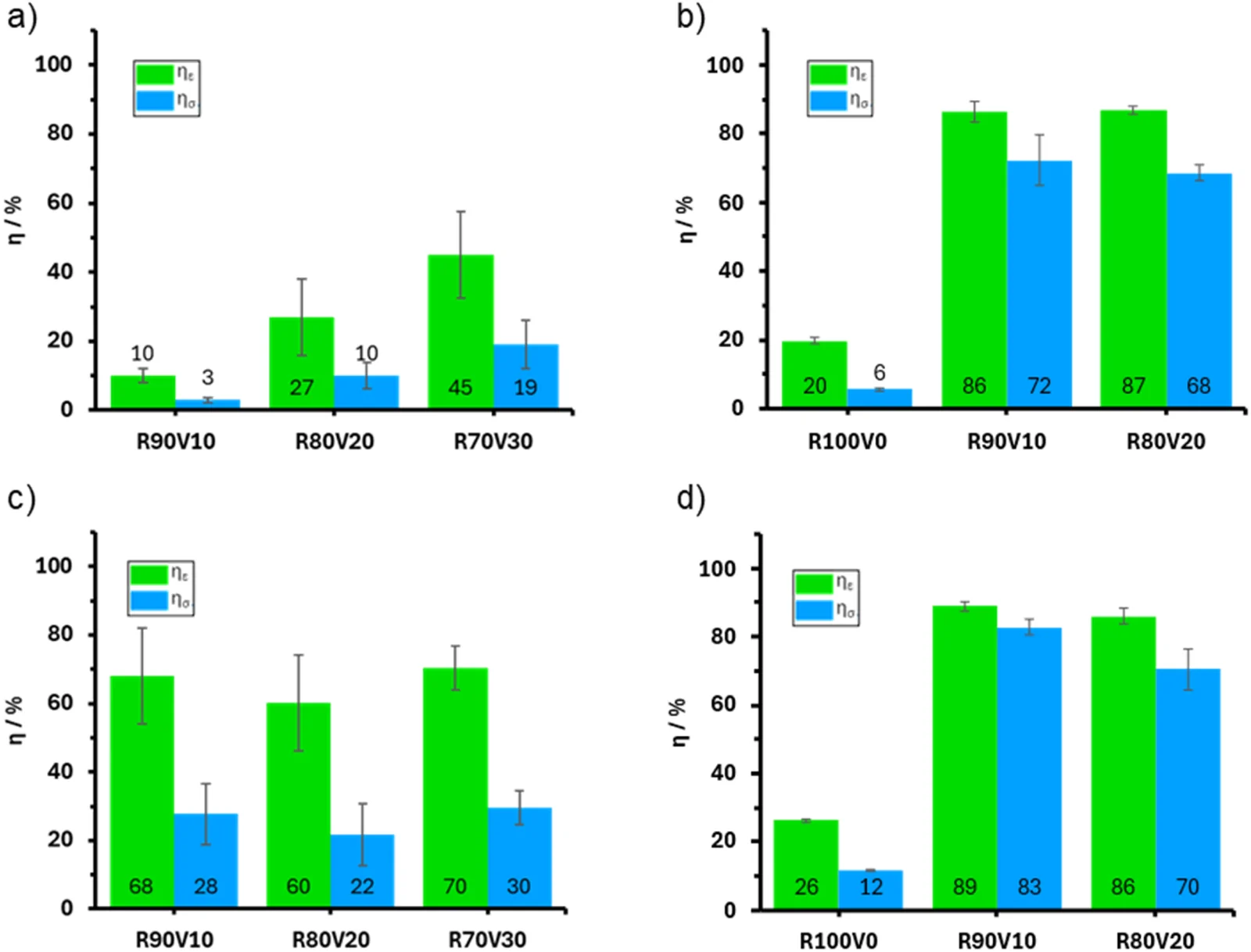
Recycling efficiency in terms of stress and strain of recycled compounds. Results for: (a) SEV-control, (b) SEV-TPP, (c) SEV-CuMA, and (d) SEV-blend. Green bars for *η*_*σ*_, blue bars for *η*_*ε*_, standard deviation in solid black line.

## Conclusions

At the end of life for most crosslinked rubber products, they will undergo a range of different processes, with some of these products ending up being disposed of in landfill sites, where the material will sit and leach off components which may damage the environment, or they may be pyrolysed which may also release harmful gases. Therefore, a complete recycling of the product requiring no additional processing significantly enhances the opportunity to use elastomers in the circular economy. Both CuMA and TPP show high levels of stability within the rubber formulations, showing minimal to no blooming. The leaching of the chemicals from the system could be understood through analysing the solvent utilised in swelling tests using such methods as chromatography to measure for copper or phosphorus traces.

Initial MDR showed that both CV-CuMA and CV-blend maintained good maximum torque recovery compared to CV-Control and CV-TPP. Small levels of reversion were seen in CV-Blend, CV-control and CV-TPP, whereas CV-CuMA showed a stable maximum torque up to 30 min. The same trends were seen in the SEV compounds. Mechanical testing of recycled compounds reported similar results between CV-CuMA and CV-Blend, showing 84% and 89% for *η*_*σ*_ and 94% and 96% for *η*_*ε*_ respectively for R80V20. CV-TPP shows little change to CV-Control. SEV compounds showed similar trends with high recycling efficiencies, however SEV-Blend and SEV-TPP showed slightly higher efficiencies than SEV-CuMA. In this case a *η*_*σ*_ and *η*_*ε*_ of 83% and 89% respectively for SEV-Blend R90V10.

As seen in previous papers, it is hypothesised that the charge on the Cu_2_ ion within CuMA acts as a potential coordination bonding site for the sulphur free radicals. In the case of TPP, the internal phosphorous atom contains a lone pair of electrons which is believed to once again provide a site for the sulphur radicals to coordinate with (Fig. 7), before the processing temperature is reached, where they then become free to react again. Previous literature suggests that TPP plays a very important role in the stabilisation of copper ions, allowing for a good collaborative blending between the two inhibitors,^18^ allowing for the high recycling efficiency with a reduced level of CuMA.

**Fig. 7:**
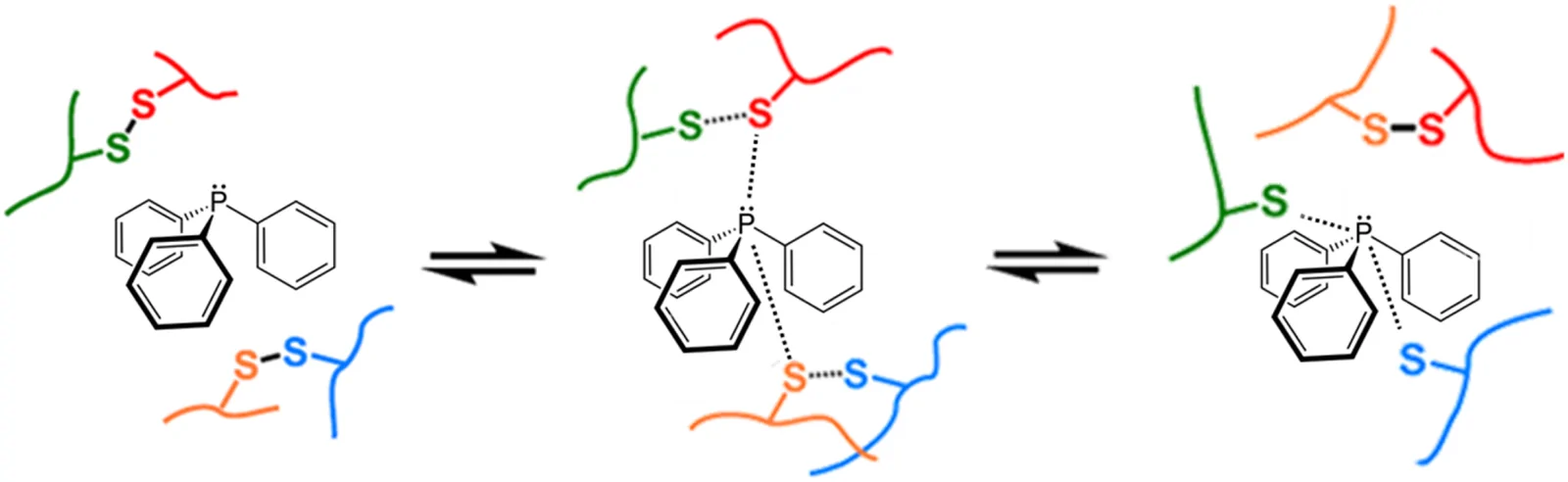
Hypothesised TPP inhibition scheme, adapted for Kaur *et al.*^13^

These hypotheses will be investigated in future work using methods such as spectroscopic probes, and post recycling crosslink rank distribution investigations. Future investigations will include understanding multiple recycling cycles as well as what role the addition of filler has on the recycling efficiencies of the system.

These findings are particularly important as it allows for similar recycling efficiencies of CV formulations and slightly higher efficiencies for SEV formulations when compared to pure CuMA at a significantly reduced cost. Between pure CuMA and the blend, there are minimal differences in the processing times, while the same total phr of inhibitors is utilised in both cases, with CuMA costing about $10.5 per gram, while the blend equates to around $2.5 per gram. There is no difference in the compounding or recycling stages for the two different systems, allowing for the estimation of a fourfold reduction in the material cost. This makes this approach to recycle compounds much more commercially viable.

## Experimental

### Materials and methods

#### Materials

Natural rubber (NR, SMR CV60 grade) was supplied by Tun Abdul Razak Research Centre (TARRC) (United Kingdom). *N*-Isopropyl-*N*′-phenyl-*p*-phenylenediamine (IPPD; 95% purity), sulphur (∼325 mesh, 99.5% purity) and *n*-cyclohexylbenzothiazol-2-sulphenamide (CBS) were purchased from Alfa Aesar. Copper(ii) methacrylate (CuMA, technical grade) was purchased from Fisher Scientific. Triphenylphosphine, was purchased from Sigma-Aldrich. Stearic acid (StA; 95% purity, reagent grade) and zinc oxide (ZnO; ≥98% purity, Light Technical grade) were purchased from VWR International Ltd.

#### Nomenclature

In the present work, CV and SEV systems were formulated and tested in different conditions before and after initial curing and subsequent recycling. The terminology adopted to refer to the formulations in specific conditions is as follows:

• Uncured compound: virgin rubber compound that has been mixed but not cured.

• Pristine-cured compound: freshly cured compound.

• Reclaimed compound: material that, after vulcanisation, was collected for recycling.

• Recycled compound: recycled cured compound.

#### Preparation of rubber samples

Rubber formulations are reported in Table 3. The compounding procedure, which for simplicity only used an open two roll mill, was as follows;

**Table 3:** Rubber formulations in phr

/phr	CV control	CV TPP	CV CuMA	CV blend	SEV control	SEV TPP	SEV CuMA	SEV blend
NR	100	100	100	100	100	100	100	100
ZnO	3.5	3.5	3.5	3.5	3.5	3.5	3	3
StA	2.5	2.5	2.5	2.5	2.5	2.5	3	3
IPPD	2	2	2	2	2	2	2	2
Sulphur	2.5	2.5	2.5	2.5	2.5	2.5	2.5	2.5
CBS	0.5	0.5	0.5	0.5	2.5	2.5	2.5	2.5
CuMA	—	—	2.47	0.6	—	—	0.62	0.16
TPP	—	2.8	—	2.1	—	0.70	—	0.47

1. Masticate NR on two-roll mill for 5 min.

2. Add StA and mix for 5 min.

3. Add ZnO and mix for 10 min.

4. Add IPPD and mix for 10 min.

5. Add CuMA, TPP or a blend and mix for 10 min.

6. Add Sulphur and CBS and mix for 10 min.

The obtained uncured compound was moulded in a manual hot press at 150 °C, applying a constant pressure of about 12 MPa, for a curing time equal to *t*_90_ as reported in SI Table 1 for CV compounds and SI Table 2 for SEV compounds, determined using Monsanto Moving Die Rheometer (MDR) 2000 with the lower die moving at 1.66 Hz.

#### Recycling of rubber samples

To recycle the pristine compounds, they were heated in the hot press for 5 min at 150 °C and immediately ground up using the simple two-roll mill until a coarse granulate estimated to be around 1 mm diameter was obtained. The material was blended with uncured rubber in the following mass fraction ratios (“R” stands for “reclaimed” and “V” stands for “virgin”): 90% reclaimed rubber with 10% virgin rubber (R90V10); 80% reclaimed rubber with 20% virgin rubber (R80V20); 70% reclaimed rubber with 30% virgin rubber (R70V30); 50% reclaimed rubber with 50% virgin rubber (R50V50).

The obtained blends were moulded in the manual hot press at 150 °C for the same time equal to the *t*_90_ of the related pristine compound in SI Table 1 for CV compounds and SI Table 2 for SEV compounds. For example: if the time to mould a pristine CV-CuMA compound was 12 min, then the time to mould CV-CuMA R80V20 was also 12 min.

#### Tensile testing

Tensile testing until failure at room temperature was carried out on each of the cured compounds. Dumbbell shape samples were cut out using an ISO-37-4 die cutter 24 hours after curing was completed. Testing was conducted using an Instron 68TM-10 machine equipped with a 2 kN load cell and pneumatic side action grips, at a rate of 1 mm s^−1^. The width (2.02 ± 0.07 mm) and the thickness (1.82 ± 0.08 mm) were measured prior to the start of each test. The target initial length, *L*_0_ (11.21 ± 1.24 mm), was measured after gripping the sample, ensuring the width of the sample remained constant in the gauge length. Uniaxial stress, *σ*, and strain ratio, *λ*, were calculated using the following eqn (1) and (2):1
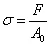
2
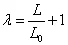
where *F* is the measured force, *A*_0_ is the initial unstrained cross-sectional area, and Δ*L* the measured elongation. Tests were repeated at least 3 times for each cured compound. A representative graph was used in each case a stress *versus* strain graph is shown.

Energy to break was found using eqn (3):3
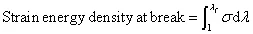
where *λ*_f_ is the strain ratio at failure, *σ* is the stress and *λ* is the strain ratio.

Recycling efficiency recycling efficiency in terms of stress, *η*_*σ*_, was calculated by comparing the ultimate tensile strength of pristine compounds and recycled compounds using eqn (4).4
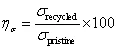


The recycling efficiency in terms of strain to break, *η*_*ε*_, was calculated by comparing the strain to break of a pristine compounds and recycled compounds using eqn (5):5
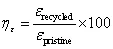


#### Equilibrium swelling

Equilibrium swelling experiments were carried out in toluene and Flory–Rehner theory was applied to calculate the crosslink density, *ν*_phy_ expressed in mol cm^−3^, of cured compounds following eqn (6):6
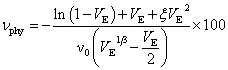
where *ν*_0_ is the molar volume of the swelling liquid, *ξ* is the Flory–Rehner interaction parameter and *V*_E_ is the volume fraction of the polymer in the mixture. A dry and rectangular shape sample for each cured compound was weighed and then immersed in a vial containing excess toluene (approximately 0.2 g of rubber in 20 mL of toluene). The vials were then left in a dark environment for 14 days. After this time, the samples were removed from the solution, the surfaces were tap dried on a white lint-free tissue and weighed again. The sample was then dried under reduced pressure to remove the solvent and then weighed a third time. The experiment was repeated three times for each cured compound. The Flory–Rehner interaction parameter for NR-toluene interaction is 0.385, while *ν*_0_ for toluene is 106.28 cm^3^. To calculate *V*_E_ the following eqn (7) is used:7
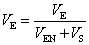
where *V*_S_ is the volume of the solvent absorbed and *V*_EN_ is the volume of elastomer network, which can be determined using eqn (8):8
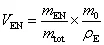
where *ρ*_E_ is the density of the cross-linked polymer, *m*_EN_ is the mass of the rubber and cross-linker used in the whole formulation, whose *m*_tot_ is the total weight and *m*_0_ is the weight of the dried sample after swelling.

## Author contributions

All authors have read and approved the final manuscript. Thomas Griggs conceived the research idea and designed the experimental methodology, performed analysis and data interpretation, drafted the initial paper and incorporated feedback from co-authors; Anureet Kaur contributed by conducting part of the laboratory experiments and collecting relevant data; Keizo Akutagawa played a key role in discussing and refining the theoretical framework and implications of the findings; Biqiong Chen and James J. C. Busfield jointly secured research funding and reviewed the data and supported the interpretation; James J. C. Busfield supervised this work and provided access to specialised equipment and facilities. All authors discussed the results and commented on the manuscript.

## Conflicts of interest

The authors declare no competing interest.

## Supplementary Material

SU-004-D6SU00080K-s001

SU-004-D6SU00080K-s002

SU-004-D6SU00080K-s003

## Data Availability

All data (MDR, GPC, DSC, tensile stress–stretch, swelling) are available from the Queen Mary University of London open access repository: https://qmro.qmul.ac.uk/xmlui/handle/123456789/130633. Supplementary information (SI) is available. See DOI: https://doi.org/10.1039/d6su00080k.
