# Supplementary material for: Optimising natural rubber recycling capability using combinations of copper(ii) ion and phosphine inhibitors
Source: RSC Sustain. 2026 Aug 18;4(10):3714–24. doi: 10.1039/d6su00080k (PMC13644683; doi:10.1039/d6su00080k)

Supplementary Information (SI) for RSC Sustainability.
This journal is © The Royal Society of Chemistry 2026

Optimising natural rubber recycling capability using combinations of copper (II) ion and phosphine inhibitors

Thomas Griggs [a], Anureet Kaur [a], Keizo Akutagawa [a], Biqiong Chen [b], and James J. C. Busfield *[a]

[a] *School of Engineering and Materials Science, Queen Mary University of London, Mile End Road, London, E1 4NS, UK*
[b] *Department of Chemistry, University of Liverpool, Crown Street, Liverpool, L69 7ZD, UK*

Supplementary Table 1: T_{90} of CV compounds

	CV-Control	CV-TPP	CV-CuMA	CV-Blend
T_{90} / min	12.88	6.79	12.06	8.24

Supplementary Table 2: T_{90} of SEV compounds

	CV-Control	CV-TPP	CV-CuMA	CV-Blend
T_{90} / min	10.04	5.07	7.65	5.92

Supplementary Figure 1: Multiple recycling events of different formulation

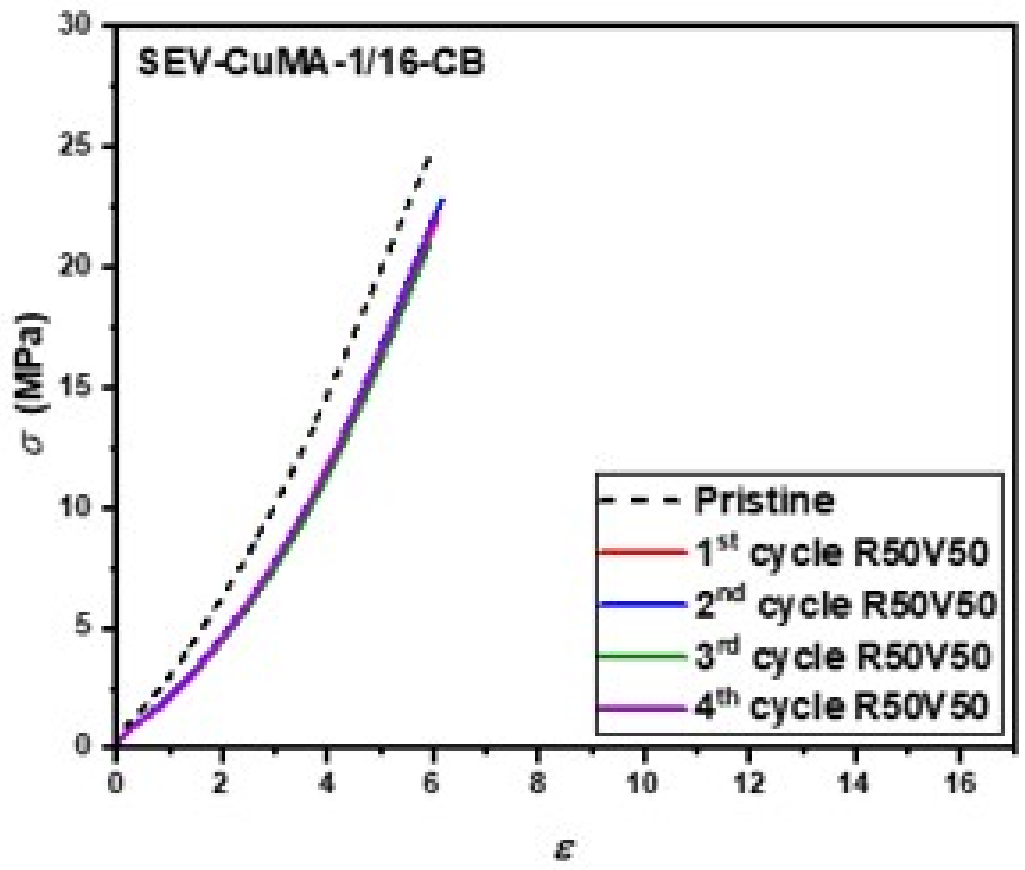

Supplementary Figure 2: Performance of different filled formulation

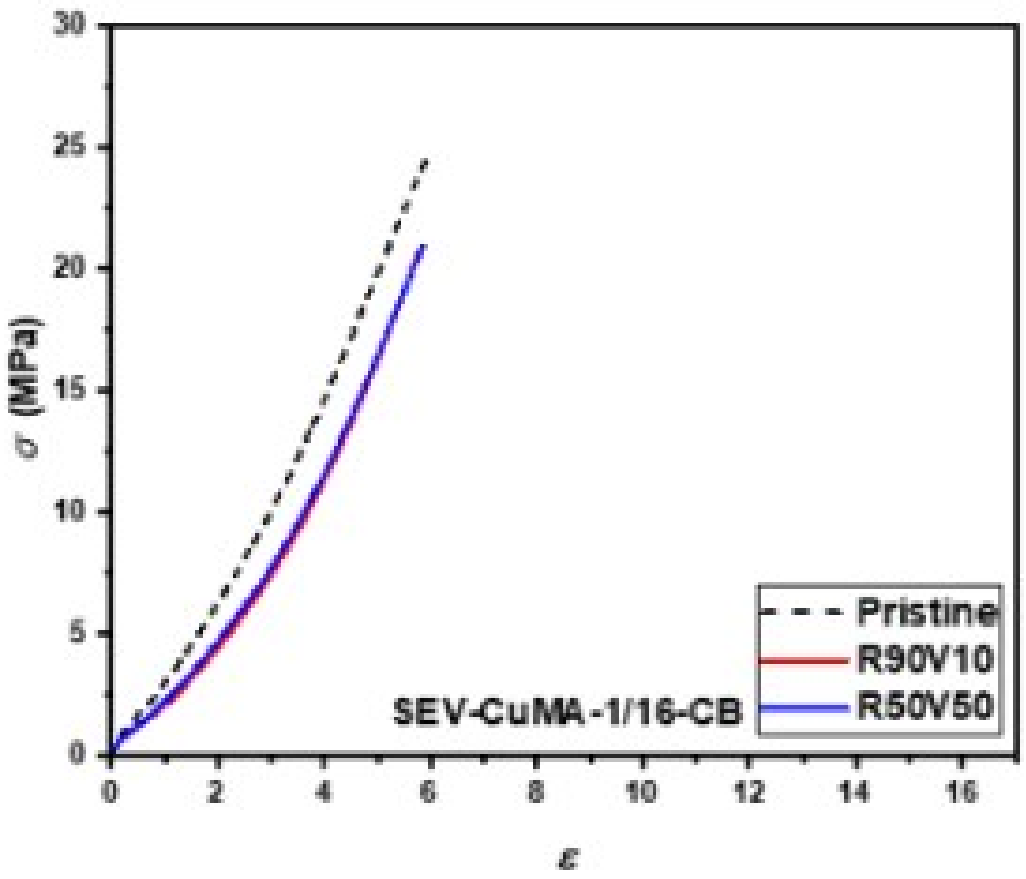

Supplement: Supplementary file 1 [file SU-004-D6SU00080K-s001.pdf]
