# Supplementary material for: Optimising natural rubber recycling capability using combinations of copper(ii) ion and phosphine inhibitors
Source: RSC Sustain. 2026 Aug 18;4(10):3714–24. doi: 10.1039/d6su00080k (PMC13644683; doi:10.1039/d6su00080k)
Supplement: Supplementary file 2 [file SU-004-D6SU00080K-s002.pdf]

This journal is © The Royal Society of Chemistry 2026

	CV-Control	CV-TPP	CV-CuMA	CV-Blend
T_{90} / min	12.88	6.79	12.06	8.24
