# Supplementary material for: Optimising natural rubber recycling capability using combinations of copper(ii) ion and phosphine inhibitors
Source: RSC Sustain. 2026 Aug 18;4(10):3714–24. doi: 10.1039/d6su00080k (PMC13644683; doi:10.1039/d6su00080k)
Supplement: Supplementary file 3 [file SU-004-D6SU00080K-s003.pdf]

Supplementary Information (SI) for RSC Sustainability.
This journal is © The Royal Society of Chemistry 2026

	CV-Control	CV-TPP	CV-CuMA	CV-Blend
T_{90} / min	10.04	5.07	7.65	5.92
